# Assessment of the coupling coordination relationship between the green financial system and the sustainable development system across China

**DOI:** 10.1038/s41598-024-62471-8

**Published:** 2024-05-21

**Authors:** Chenggang Li, Youhui Bao, Yingjie Li, Mu Yue, Liang Wu, Yufeng Mao, Tingzhang Yang

**Affiliations:** 1 School of Big Data Application and Economics, Guizhou University of Finance and Economics, Guiyang, 550025 China; 2 Guizhou Institute of Applied Statistics, Guizhou University of Finance and Economics, Guiyang, 550025 China; 3 Natural Capital Project, Stanford University, Stanford, CA 94305 USA; 4 School of Physical and Mathematical Sciences, Nanyang Technological University, Singapore, 637371 Singapore; 5 School of Economics and Management, Guizhou Normal University, Guiyang, 550025 China; 6 Guizhou Modern Urban Rural Economic Development Research Institute, Guiyang, 550025 China

**Keywords:** Green finance, Sustainable development system, Coupling coordination, Environmental sciences, Environmental social sciences

## Abstract

Green finance (GF) is recognized as a key driver of sustainable development. While existing studies have extensively discussed the relationship between GF and the Sustainable Development Goals (SDGs), few have explored the coupling coordination relationship between GF and SDGs. In this paper, we use data from thirty Chinese provinces (municipalities and autonomous regions) from 2008–2021 to examine the degree of coupling coordination development (CCD) between GF and the SDGs systems using the CCD model. We find that most SDGs and their sub-goals exhibit a significant upward trend, except for SDG8, 14–16. GF presents a fluctuating upward trend, with a significant decline in 2010 and 2019. The CCDs between GF and SDGs and their sub-goals generally show an M-shaped upward trend in most regions, with most of them experiencing a synchronous decline in 2011–2012 and 2019. In the analysis of regional heterogeneity, the eastern region performs better in SDG8–9, the central region performs better in SDG3, 14–15, while the western region performs better in SDG7. This paper provides empirical evidence for a further in-depth understanding of the relationship between GF and SDGs, which can contribute to advancing GF development and the SDG process.

## Introduction

At the United Nations Sustainable Development Goal Summit in 2015, 193 member states of the United Nations formally proposed 17 Sustainable Development Goals. The goals aim to adopt a systematic and comprehensive approach to solve development problems in the three dimensions of society, economy and environment from 2015 to 2030, and to promote economic and social transitions towards sustainable development. In order to promote sustainable development, financing methods such as green finance^1–3^, climate finance^4,5^, and green funds^6^ have been proposed internationally. Countries around the world generally believe that green finance is an interdisciplinary field of finance and the green economy, aimed at combining financial behavior with environmental protection, supporting environmental improvement, addressing climate change, and conserving resources^7,8^. Compared to traditional financial activities, green finance places more emphasis on the harmonious and balanced development of economic activities and the ecological environment^9^, effectively alleviating environmental degradation, climate change and poverty^10,11^, promoting sustainable energy development^12–14^, and ultimately achieving the SDGs. In COP27, countries agreed to establish a “Loss and Damage” fund to support vulnerable countries facing climate disaster, which was a breakthrough for both green finance and climate governance. The final agreement emphasized the necessity of investing 4–6 trillion dollars annually in renewable energy up until 2030.

Green finance sprouted in 1974 in Federal Germany, where the world's first policy-based environmental bank was established, introducing the idea of environmental protection into the operating guidelines of financial institutions. China first proposed the use of leverage to combat environmental pollution in 1981, and the environmental problems faced in economic development gave rise to the prototype of green finance in China. Subsequently, China's green finance has developed steadily, and in 2016 China added green finance to the G20 issues. In the same year, the People's Bank of China, the Ministry of Finance and seven other departments jointly issued the Guidance on Building a Green Financial System, marking China's emergence as the first country in the world to have a relatively complete green financial system. China has set up seven provinces (autonomous regions) and ten pilot zones for green financial reform and innovation since 2017. As a leading country in international cooperation on green finance, China has pushed green finance to become a global issue, promoted the development of green finance along the Belt and Road, continued to play an important role in international cooperation, and made positive efforts to address climate change and promote sustainable development.

We introduce the coupling coordination degree model (CCD) to assess the complex interactions between green finance and the SDGs. Coupling refers to the phenomenon where two or more elements or systems influence each other through interaction, emphasizing the development from disorder to order among systems. Coordination refers to the elements or systems being in harmony and working well together, emphasizing the narrowing of the gap between the elements as the system moves and develops. Coupling coordination emphasizes the degree of coordination between the internal elements of the system that interact and couple with each other in the process of system movement and development, which determines the trajectory of the integration of the green finance-sustainable development goal system from disorder to order.

A growing body of research has explored the relationship between green finance and SDGs, mainly focusing on the following aspects: (1) the relationship between green finance and individual sub-goals, including poverty, infrastructure, energy security, environmental protection, agricultural development, education, and economic development, among others^11–21^. (2) The impact of green finance on SDGs, including its effects on environmental sustainability, energy sustainability, agricultural sustainability, education sustainability, economic sustainability and other Sustainable Development Goals^2,13,21,22^. (3) The indirect channels through which green finance affects the SDGs. Green finance can promote green technology innovation and the development of green enterprise, accelerate the transition from traditional energy consumption, mitigate environmental pollution and climate change, and regulate the relationship between innovation and the energy environment to achieve the SDGs^15,23,24^. Previous studies have primarily focused on the mechanisms and effect sizes of green finance on the SDGs, with the object of study mostly being the overall SDGs or one of the 17 SDG subgoals. However, fewer studies have examined the coupling and synergy between green finance and the SDGs and their sub-goals. There is thus a lack of research on the coupling and coordination relationship between green finance and the sub-goals of the SDGs.

To address the significant knowledge gap, we explored the coupling coordination relationship and the coordinated development effect between China's green finance and the SDGs. We delve into the development of green finance and the SDGs in China, examining coupling and synergistic development across both time and space dimensions, and provide theoretical advice for further constructing green finance and the SDGs in China. Since the reform and opening up, China's economy has developed rapidly, becoming the world’s second largest economy^25^, as well as the largest energy consumer and carbon emitter^26^. Therefore, transitioning towards sustainable development while developing the economy is one of the biggest challenges faced by China and other regions globally. Meanwhile, China's green financial development is at the forefront globally, with the scale of China's green credit ranking first in the world and green bond issuance maintaining the second place in the world. Therefore, assessing the coupling coordinated development relationship between China's green finance and the SDGs can provide valuable information for the green finance and sustainable development of countries worldwide.

In this study, we addressed the following questions: (1) What is the level of development of green finance and SDGs in China? (2) What is the coupling coordination relationship between China's green finance and the SDGs? (3) Is the coupling coordination development of green finance and SDGs different in China’s different regions? To answer these questions, we first used the entropy method to calculate the green financial system index and the scores of each SDG sub-goal. Then we constructed the coupling coordination degree model to assess the coupling coordination relationship between China’s green finance and the SDGs sub-goals and explore the coupling synergy between green finance and each SDG sub-goal from the perspective of sustainable development. We find that SDGs and most of the sub-goals have a clear upward trend, and the green finance fluctuates with upward development in each province, declining significantly in 2010 and 2019. The coupling coordination degree of green finance with SDG and its sub-goals shows a largely M-shaped upward trend in most regions, with most of them showing a synchronized decline in 2010–2011 and 2019. The eastern, central and western regions perform differently on different sub-goals.

## Models and data

**Figure 1:**
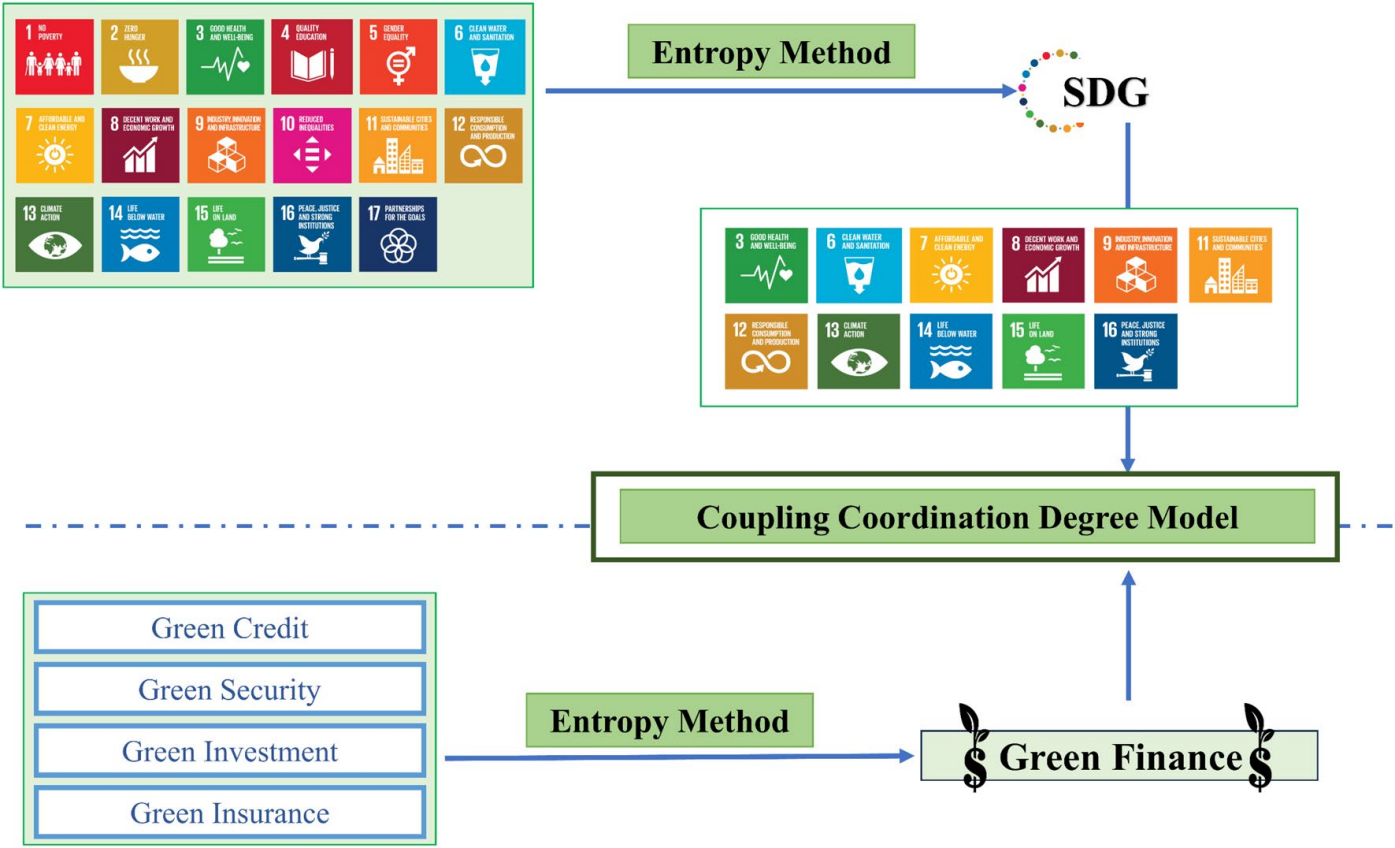
Methodological flowchart.

We adopt the entropy value method to establish the index system, and then use the coupled coordination degree model to carry out the empirical test. The specific method flowchart is shown in Fig. 1.

### Construction of the indicator system

1. Sustainable development goals. Referring to *The 2030 Agenda for Sustainable Development of the United Nations* and the study by Xu et al.^25^, we established an indicator system based on the 17 goals of sustainable development and data availability, which includes indicators for poverty eradication, hunger eradication, quality education, gender equality, clean water, etc. The specific indicators are shown in Supplementary Table 1.
2. Green finance. Green finance, referring to the studies of Zhou et al.^27^, Gao and Zhang^28^, Shi and Shi^25^, we establish a system of green finance indicators from four dimensions: green credit, green security, green investment, and green insurance. Green credit refers to a series of policies, institutional arrangements and practices that utilize credit means to promote energy conservation and emission reduction. Compared with high energy-consuming enterprises, green credit is more inclined to provide loans to environmentally friendly enterprises, and this inclination will inevitably affect the interest expenses of enterprises in the corresponding industry. This paper adopts the percentage of interest expenses of high energy-consuming enterprises as a green credit measurement index. Green security is a securities system and economic behavior under the premise of environmental protection and sustainable development through the securities market to promote the coordinated development of economic, society and the environment. This paper adopts the proportion of market capitalization of high-energy-consuming industries in total market capitalization to measure. The lower the proportion indicates the better the development of green securities. Green insurance is a figurative name for environmental liability insurance, which refers to the liability insurance that takes the damage compensation and governance responsibility assumed by the insured for polluting the environment as the subject of the insurance. The scope of green insurance in China can include liability insurance for oil pollution damage of ships, agricultural insurance, forest insurance, and other liability insurances involving ecological and environmental elements. In this paper, given the availability of data, only agricultural insurance is involved. The specific indicators are shown in Supplementary Table 2.

### Data

Considering data availability (we did not use the data of Tibet, Hong Kong, Macao, and Taiwan), we selected panel data from 30 provinces (municipalities and autonomous regions) of China over 2008–2020. The data were collected from various sources, including the *China Statistical Yearbook*, *China Statistical Yearbook of Education*, *China Statistical Yearbook of Population and Employment*, *China Statistical Yearbook of Energy*, *China Statistical Yearbook of Environment*, *China Statistical Yearbook of Marine Economy*, *China Marine Statistical Yearbook*, *China Statistical Yearbook of Science and Technology*, *China Industrial Statistical Yearbook*, *China Fiscal Yearbook*, *China Statistical Yearbook of Health*, *China Procuratorial Yearbook*, the work reports of the people’s procuratorate of each province (municipality and autonomous region), the statistical yearbook of each province (municipality and autonomous region), the database of the National Bureau of Statistics, the CSMAR database, the CEADs database, and the *Fourth National Economic Census*. There are missing values in the data for a few variables, but at most not more than 3 years, so for missing values we use interpolation method to fill in the blanks.

We used the entropy method to calculate the score of GF, SDGs and 17 SDG sub-goals indicators. GF and the 17 SDG sub-goals were derived using the entropy method respectively. The overall SDG was derived using the entropy method again for the resulting 17 sub-goals.

The steps of the entropy method are as follows:

Firstly, we standardized the collected data according to Eqs. (1) and (2) respectively.

1 $$ z_{ij} = \frac{{x_{ij} - \min (x_{j} )}}{{\max (x_{j} ) - \min (x_{j} )}} $$

2 $$ z_{ij} = \frac{{\max (x_{j} ) - x_{ij} }}{{\max (x_{j} ) - \min (x_{j} )}} $$

where Eq. (1) is the standardization of positive indicators and Eq. (2) is the standardization of negative indicators. *z*_*ij*_ denotes the value of indicator i in the j-th province after standardization, *x*_*ij*_ denotes the value of indicator i in the j-th province (j = 1, … , n, i = 1, … , m), max(x_j_) and min(x_j_) denote the maximum and minimum values for the j-th province, respectively. After that we calculate the weight of the indicator $p_{ij} = \frac{{z_{ij} }}{{\sum\nolimits_{i = 1}^{m} {z_{ij} } }}$. In order to avoid p_ij_ = 0, we add 0.0000000000001 to the calculated weight, as shown in Eq. (3).

3 $$ p_{ij} = \frac{{z_{ij} }}{{\sum\nolimits_{i = 1}^{m} {z_{ij} } }} + 0.0000000000001 $$

Second, the entropy value of the j-th indicator and the weight of each indicator are calculated as shown in Eqs. (4) and (5).

4 $$ e_{j} = - k\sum\limits_{i = 1}^{m} {p_{ij} \ln (p_{ij} )} $$

5 $$ w_{j} = \frac{{1 - e_{j} }}{{\sum\nolimits_{i = 1}^{m} {1 - e_{j} } }} $$

where *k* > 0, *e*_*j*_ > 0. The constant k is related to the sample number m and estimated by *k* = *1*/ln(*m*), then 0 ≤ e_j_ ≤ 1.

Third, the comprehensive score for each sample is calculated using Eq. (6).

6 $$ S_{i} = \sum\limits_{i = 1}^{n} {w_{j} \times z_{ij} } $$

### Coupling coordination degree model (CCDM)

The coupled coordination degree model is a widely used tool to analyze the level of interaction and coordinated development between different systems or subsystems. It is now widely used in the fields of economics, environment, social development, and other multidisciplinary fields. In economics, the model is primarily used to analyze the level of coordinated development among multiple systems such as the environment, economy and society, for instance, measuring the interaction between ecology and economy^20,29,30^. Additionally, it is widely used in the research of green finance^31,32^ and sustainable development^33,34^.

Seven ministries and commissions, including the People's Bank of China, define green finance as the economic activities that support environmental improvement, climate change response and the economical and efficient use of resources. This means providing financial services for project investment and financing, project operation and risk management in the fields of environmental protection, energy conservation, clean energy, green transportation and green buildings, and so on. This definition is highly overlaps with SDGs 6–7 and SDGs 12–15 of the sustainability goals, and the financial services provided by green finance will inevitably create synergies with the realization of the above goals. The transformation of environment and climate will have an impact on human living conditions and health, which means green finance is indirectly linked to SDG3 and SDG11. However, green finance still has a long way to go and requires more specialized human and material resources, which provides opportunities for employment and also promotes green science and technology innovation, green industry development, and the high-quality development of the economy, aligning with the objectives of SDG8–9^20^. In summary, green finance is closely related to the SDGs, and studying the interactions between the two systems is conducive to the coordination development of both systems. In this study, we use the Coupling Coordination Degree Model to measure the coordination development of GF and SDGs.

Coupling is primarily a phenomenon in which two or more systems affect each other through traceable interactions. The coupling degree model is shown in Eq. (7).

7 $$ CA_{it} = \sqrt {\frac{{GF_{it} \times A_{it} }}{{\left( {\frac{{GF_{it} + A_{it} }}{2}} \right)^{2} }}} $$

where, CA_it_ denotes the coupling of SDG and SDGn (n = 3, 6, … 9, 11, … 16) with GF in year t of the i-th province (municipality, autonomous region).

To determine the degree of coordination between the systems, the coupling degree can only reflect the mutual influence between the systems. It is difficult to reflect the level of development of each system itself, so it is also necessary to further judge the coupling and coordination relationship between the systems. The coupling coordination degree model is shown in Eq. (9):

8 $$ TA_{it} = \frac{{GF_{it} + A_{it} }}{2} $$

9 $$ DA_{it} = \sqrt {CA_{it} \times TA_{it} } $$

where, *DA*_*it*_ denotes the degree of coupling coordination between GF and SDG or *SDGn* (n = 1,2,3 … 17) in year t of the i-th province (municipality, autonomous region). *TA*_*it*_ is the combined evaluation score of the 2 systems. *A*_*it*_ denotes SDG and *SDGn* (n = 3, 6, … 9, 11, … 16) in year t of the i-th province (municipality, autonomous region).

### Classification of coupling coordination types

In this paper, we use the uniform distribution method to divide the coupling coordination degree level of GF and SDGs, and the specific division criteria are shown in Table 1.

**Table 1:** Criteria for categorizing the degree of CCD between the GF system and the SDG system.

Coupling coordination degree (CCD)	Nexus
0.0–0.1	Strong trade off
0.1–0.2	
0.2–0.3	Weak trade off
0.3–0.4	
0.4–0.5	Interaction
0.5–0.6	
0.6–0.7	Weak synergy
0.7–0.8	
0.8–0.9	Strong synergy
0.9–1.0	

## Results

### Green finance

**Figure 2:**
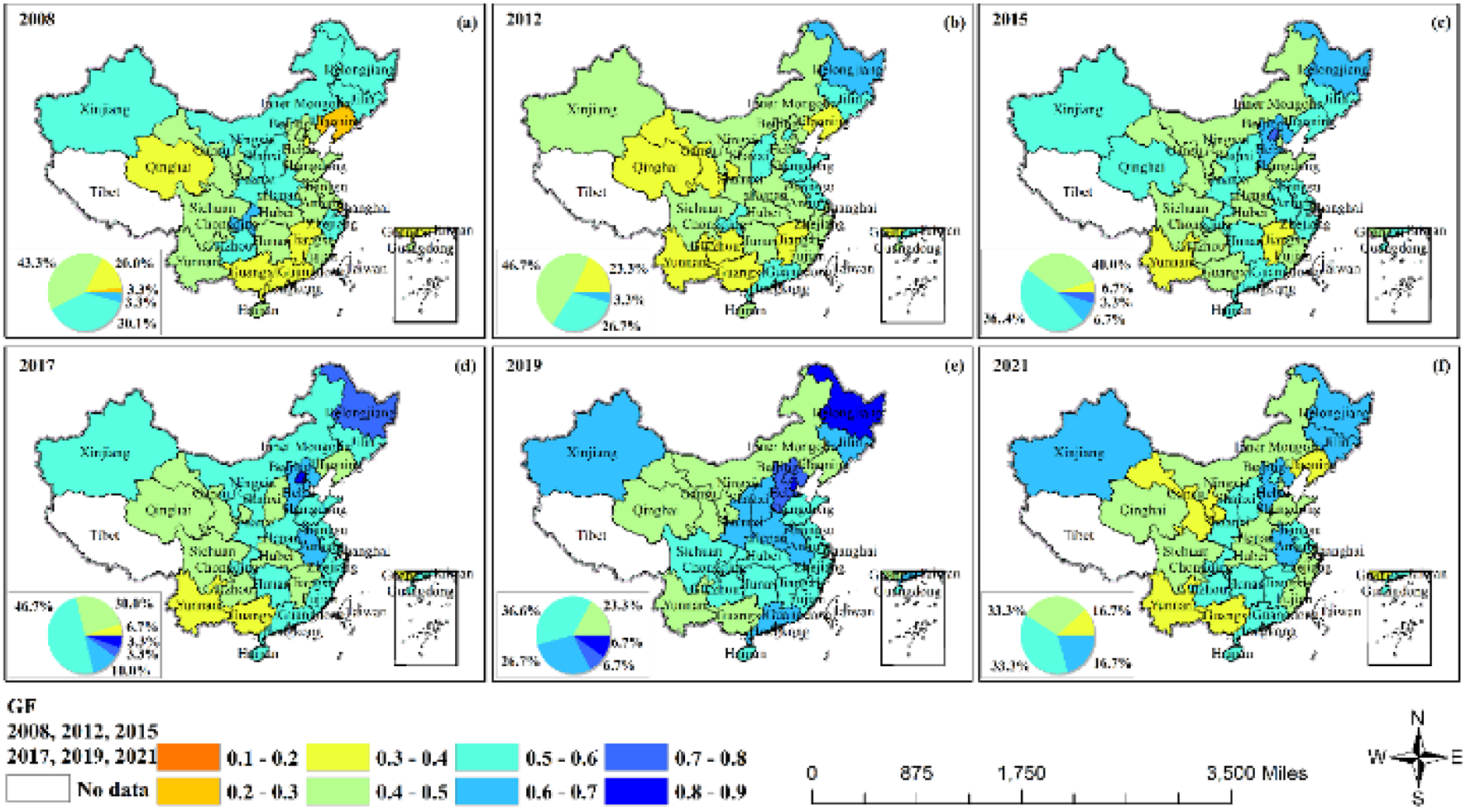
Green finance.

The nation's green finance index showed an upward trend before 2019 and a downward trend after 2019. In 2008, Liaoning’s GF index was less than 0.3, which was the lowest value of the nation's GF index, with only Chongqing exceeding 0.6. In 2019, the nationwide value was more than 0.4, and 76.7% of the provinces' values were more than 0.6 (Fig. 2 and Fig. S1). It declined markedly after 2019, with only 16.7% of the nation's provinces' values exceeding 0.6, and 16.7% of provinces being less than 0.4. Geographically, the development of green finance in north China showed a declining-rising-declining trend, while northeast, east, and central China steadily increased until 2019 and then declined.

### Sustainable development goals

**Figure 3:**
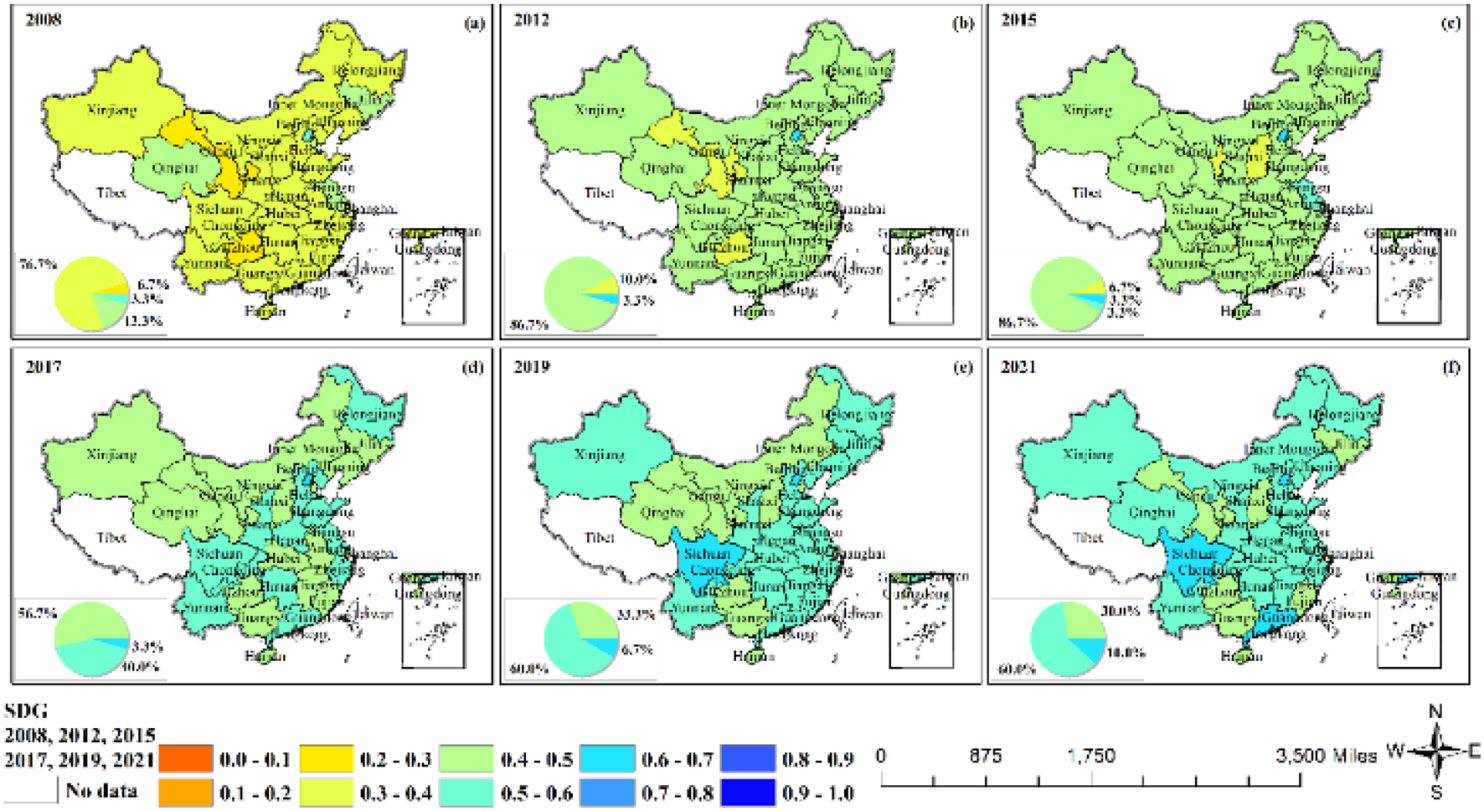
Sustainable development goals.

Numerically, the development level of SDG generally lagged that of GF but was on an upward trend as a whole: the average value in 2008 was 0.37, and the average value in 2021 rose to 0.53. Geographically, Beijing's development level of SDGs was more prominent, consistently maintaining a certain gap with other provinces. Additionally, the growth of SDG showed a development trend from east to west and from south to north (Fig. 3 and Fig. S2).

Figure S3 shows the trends of SDG1–9 in China. The SDG1 of each province showed a fluctuating and rising development trend, with a large difference in the level in the early stage, gradually narrowing in the later stage. Beijing's SDG1 was significantly higher than that of other provinces. Sichuan had the biggest increase, from 0.09 in 2008 to 0.78 in 2021. SDG2 performed poorly in general, with the vast majority of provinces having values less than 0.5, but all showing a steady upward trend. Among them, Qinghai and Hainan were significantly weaker than the other provinces. SDG3 performed better overall and basically rose year by year, with the exception of Qinghai and Xinjiang, whose development significantly lagged behind that of the other provinces, with the rest of the provinces rising in the range of 0.5–0.8. The SDG4 of provinces and cities fluctuated within the range of 0.3–0.0.65, with large fluctuations and differing development trends. The SDG5 of most provinces fluctuated slightly within the range of 0.45–0.5, with no obvious upward or downward trend. Among them, Xinjiang, Tianjin and Beijing had the most obvious fluctuations. The development trend of SDG6 in each province was relatively uniform, with most showing high-speed development in 2008–2010 and 2015–2016, low-speed development in 2011–2015 and 2016–2019, and sudden decline in 2010–2011 and 2019–2020. Among them, Jiangsu and Guangdong performed significantly better than other provinces. SDG7 in most provinces rose at a high speed in 2008–2009, followed by a significantly weaker development. SDG8 for most provinces remained flat during 2008–2021 and fell sharply in 2020. Among them, Beijing performed significantly better. The SDG9 of each province showed a steady upward trend and was divided into three echelons in terms of value from high to low: Beijing far outstrips the remaining provinces, Ningxia and Shanxi lagged significantly behind, and the rest of the provinces developed similarly.

Figure S4 shows the trends of SDG10–17 in China. The SDG10 performance of most provinces was relatively similar: it declined in 2010 and 2020, and remained basically stable from 2011 to 2019. SDG11 in the provinces showed a fluctuating upward trend. SDG12 in some provinces was flat in the early part of the year and rose in the later part of the year, showing a J-shaped upward trend. SDG13 of some provinces remained flat, while some provinces fluctuated more. Since ocean data for most provinces are missing, the corresponding SDG14 data overlapped on a line, and the difference in SDG14 among neighboring provinces was obvious. SDG15 fluctuated within the range of 0.5–0.7 in most provinces, with most provinces experiencing decreasing values in 2009 and 2019. SDG16 fluctuated significantly across provinces, with some provinces showing fluctuating decreases and a significant decrease in 2019. The SDG17 of each province showed a U-shaped development trend, with Beijing, Tianjin and Shanghai performing significantly worse.

### Coupling coordination degree

**Figure 4:**
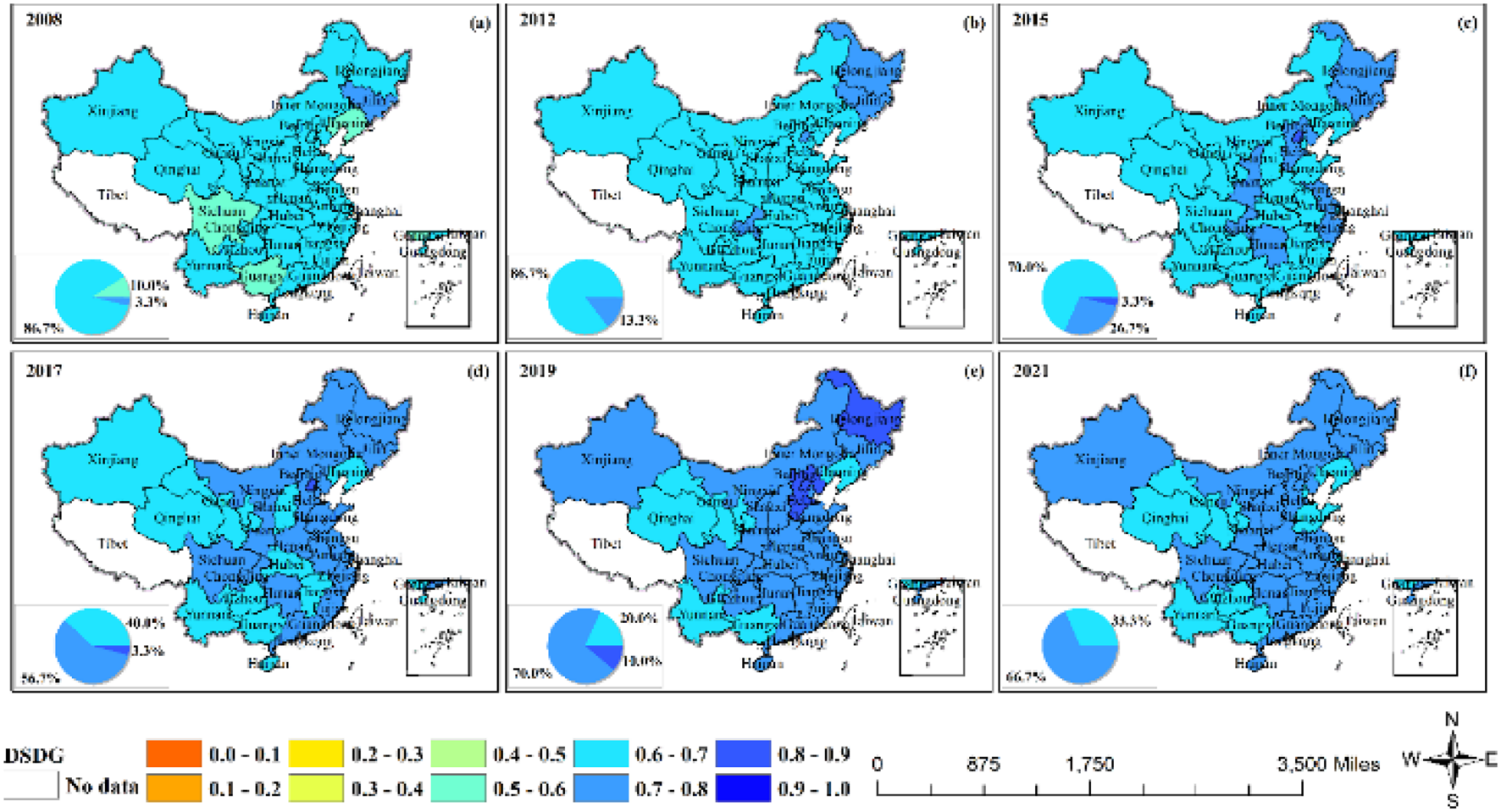
The coupling coordination degrees of GF and SDG.

The GF had a high level of CCD with the overall SDG, with 86.7% of the regions having a CCD level above 0.6 in 2008, reaching primary coordination (weak synergy) (Fig. 4 and Fig. S5). Subsequently, the coupling coordination level of each region increased steadily from east to west, and 66.7% of the regions had a value over 0.7 in 2021, reaching intermediate coordination (weak synergy). Among them, Beijing, Chongqing, Heilongjiang, and Jilin were more prominent. A small number of regions showed a downward trend after 2019. The outbreak of COVID-19 caused a huge impact on the global economy, and green finance as a part of economic activities, was also inevitably affected, with the green finance index declining after 2019 (Fig. S1). According to existing studies, the epidemic only slowed down the process of achieving the SDGs^35^. Our measurements also indicate that the SDG index was still on an upward trend after 2019 (Fig. S2). The two systems showed opposite trends, resulting in a decrease in coupling coordination.

**Figure 5:**
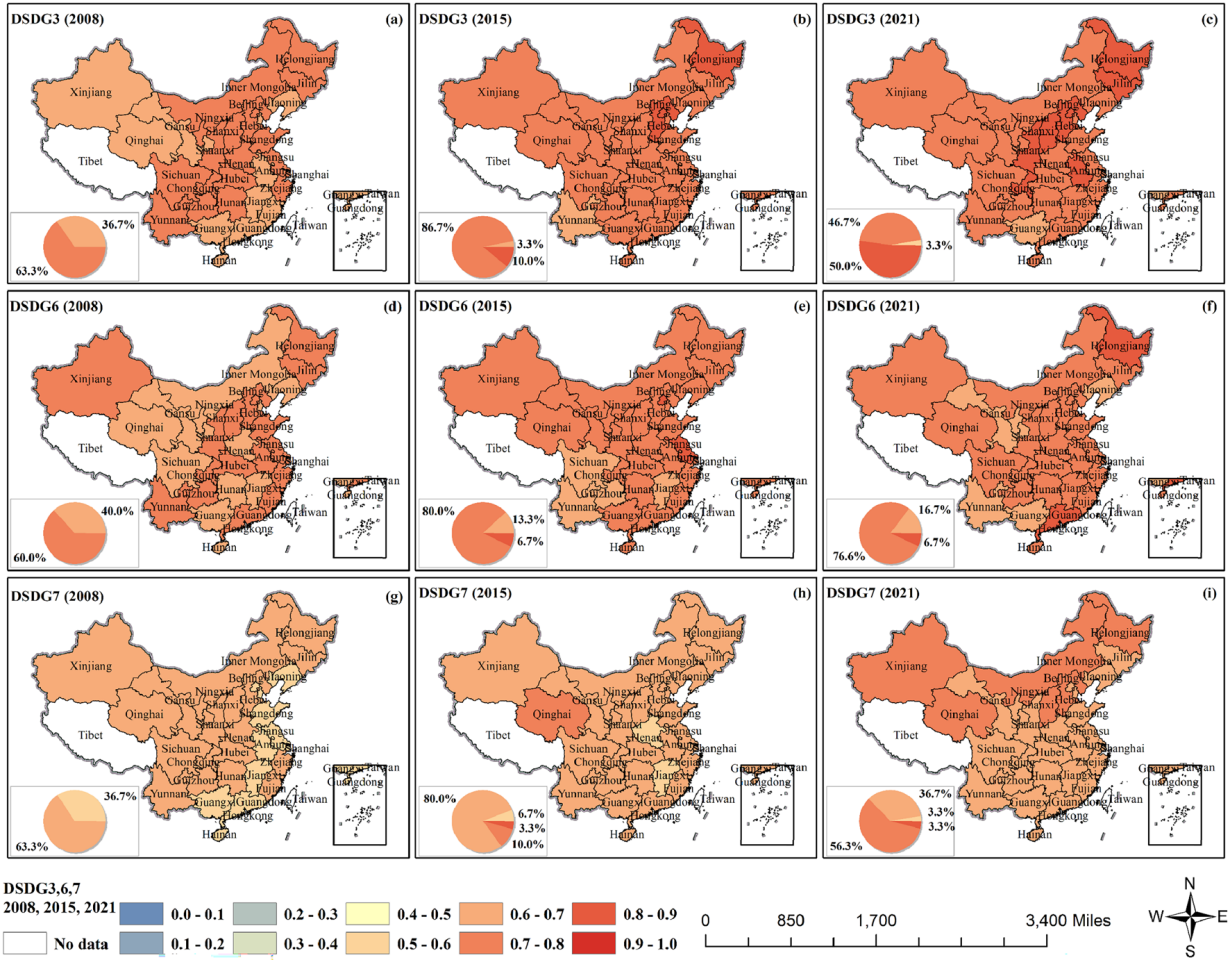
The coupling coordination degrees of GF and SDG3,6,7.

The CCD of GF and SDG3,6,7 showed a significant increasing trend (Fig. 5 and Fig. S6). GF invests money in projects and companies that use clean energy and reduce environmental pollution, which can mitigate pollution of air, land and water, and the reduction of environmental pollution has a direct impact on public health (SDG3). The CCD of GF and SDG3 (health and well-being) was better, and the values had all reached primary coordination in 2008, with 63.3% of the regions reaching intermediate coordination. After that, the CCD of most regions stayed stable or gradually rose, with 50.0% of the provinces reaching over 0.80 in 2021 to achieve good coordination, which was in the overall weak synergy and strong synergy. GF funding leads to reduced water pollution, and reduced water pollution leads to improved public health and improved drinking water quality (SDG6). The CCD between GF and SDG6 (clean water) had all reached primary coordination in 2008, with 60.0% of the provinces reaching intermediate coordination, followed by a significant increase in the central region provinces. Then, central region provinces increased significantly, with 76.6% of the provinces in the country reaching intermediate coordination and 6.7% reaching fine coordination in 2021. GF financing for renewable energy sources such as wind, solar and biomass can enhance energy security and sustainability (SDG7). The CCD between GF and SDG7 (clean energy) has reached grudging coordination across the board in 2008. Then it gradually rose, most notably in northeast, north, and northwest China, with all regions reaching primary coordination and 20% of provinces reaching intermediate coordination in 2021. Overall interaction gradually turned to weak synergy.

**Figure 6:**
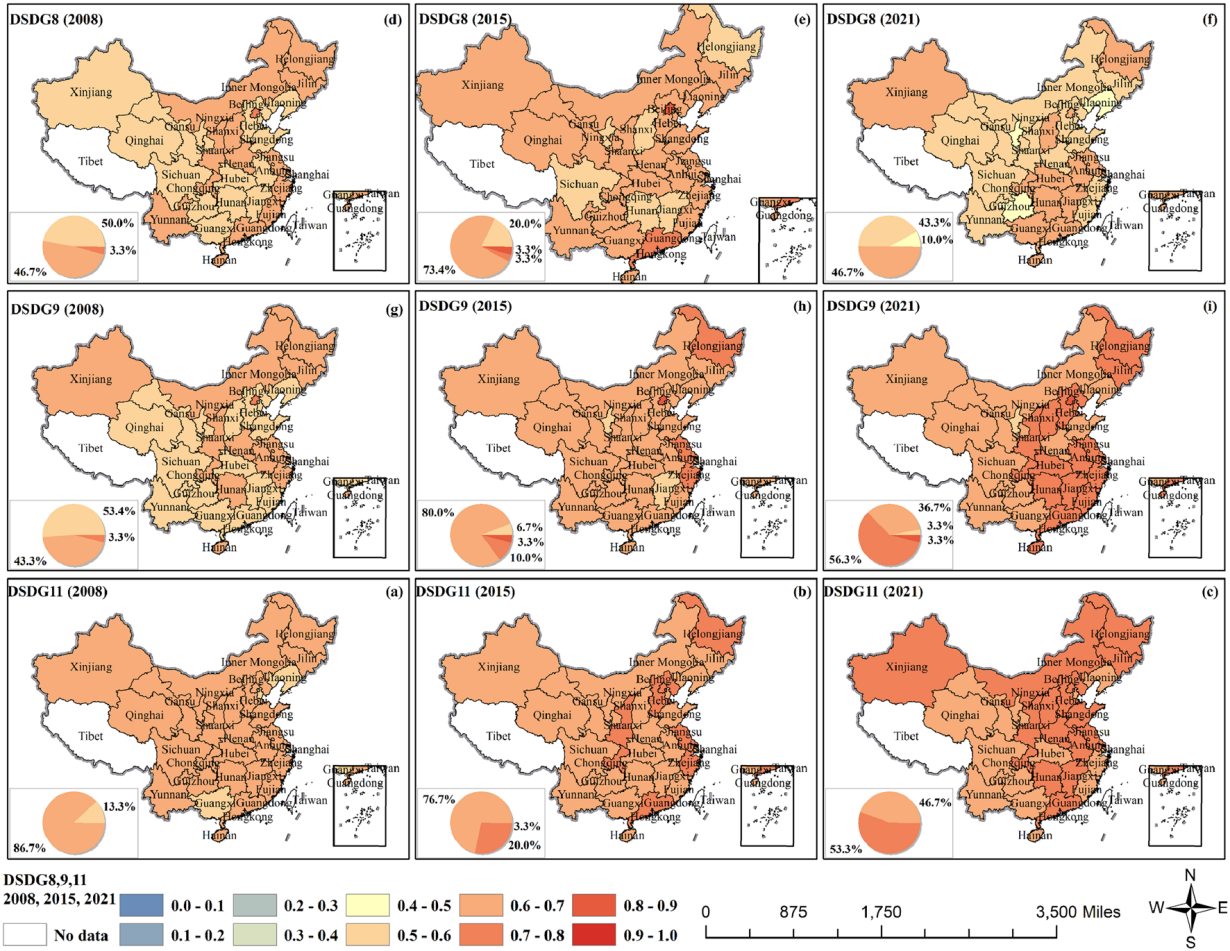
The coupling coordination degrees of GF and SDG8,9,11.

The CCD of GF and SDG8,9,11 showed a significant and synchronized decline in both 2010 and 2019 (Fig. 6 and Fig. S6). Along with the development of green finance, a series of green industries and green innovative technologies have been bred in the world, leading to the development of infrastructure and the creation of more employment opportunities (SDG8 and SDG9). The CCD of GF and SDG8 (decent work) remained generally stable, fluctuating up and down between interaction and weak synergy. In 2008, all provinces reached grudging coordination, with 46.7% of provinces reaching primary coordination, and Beijing reached intermediate coordination. The CCD of most regions decreased in 2010 and 2019. However, in 2021, only 43.3% of the provinces reached grudging coordination, and 10.0% of the provinces were even in near maladjustment. The CCD of the GF and SDG9 (industrial innovation) maintained a steady growth, and the interaction gradually rose to weak synergy. Only 43.3% of the provinces reached grudging coordination in 2008. After that, the values gradually increased except for the decline in 2010 and 2019, most noticeably in the southeast region. 56.3% of provinces reached intermediate coordination in 2021, with most of them concentrated in central and eastern China. GF can finance public transportation and energy-efficient buildings, support urban green transitions and reduce carbon footprints to create sustainable cities and communities (SDG11). The development of CCD between GF and SDG11 (perpetual community) was in a good situation, with 86.7% of provinces reaching primary coordination in 2008. Then the value gradually increased in the eastern region, and the interaction turned to weak synergy. In 2021, 53.3% of provinces reached intermediate coordination.

**Figure 7:**
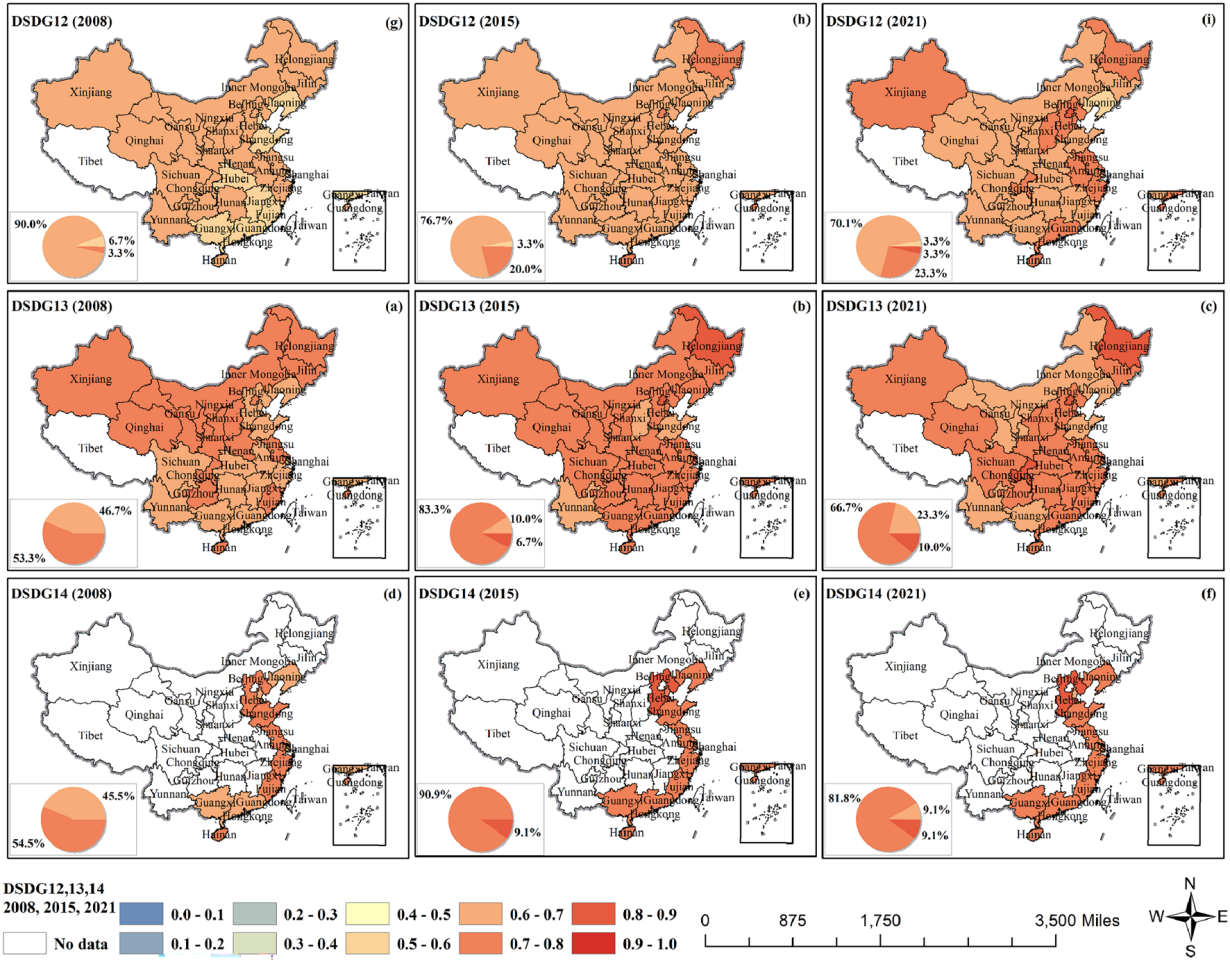
The coupling coordination degrees of GF and SDG12–14.

The CCD of GF and SDG12–14 showed a significant and synchronized decline in both 2010 and 2019 (Fig. 7 and Fig. S7). Green finance guides businesses and industries toward cleaner, more environmentally friendly and sustainable development (SDG12). The CCD between GF and SDG12 (perpetual supply and demand) was slightly weaker than that between SDG10 and SDG11. 80.0% of provinces reached primary coordination in 2008, with a slower increasing trend of values, and 36.7% of provinces reached intermediate coordination in 2021 with a more dispersed distribution. All of them were in weak synergy. The CCD between GF and SDG13–15 was relatively good, and they were all in weak synergy in general, with a significant and synchronized decline in both 2010 and 2019. GF can channel more funds into projects for clean energy development and carbon emission reductions, which directly support global efforts to combat climate change (SDGG13). In 2008, the CCD between GF and SDG13 (climate action) reached intermediate coordination in 53.3% of the provinces, with most of them concentrated in the northern region. Then the values in each region have increased. In 2021, 66.7% of provinces reached primary coordination, and 10% of provinces reached intermediate coordination, but the value in north China decreased to primary coordination.

Due to the large amount of missing relevant marine data from non-sea-front provinces, only data from 11 costal provinces were selected for further study for the sake of accuracy of the study. Within GF there is blue finance, which focuses on sustainable projects in oceans and waters such as ecological conservation, sustainable fisheries, marine energy development, etc., to protect underwater ecosystems and promote their sustainable use (SDG14). The CCD of GF and SDG14 (marine environment) reached primary coordination in most of the regions from 2008, and then it remained steadily. In 2021, more than 90.0% of provinces reached intermediate coordination, and even 9.1% of provinces reached fine coordination. CCD will be higher in the northern provinces. Among the 11 provinces, Hebei has the best degree of coupling synergy between GF and SDG14.

**Figure 8:**
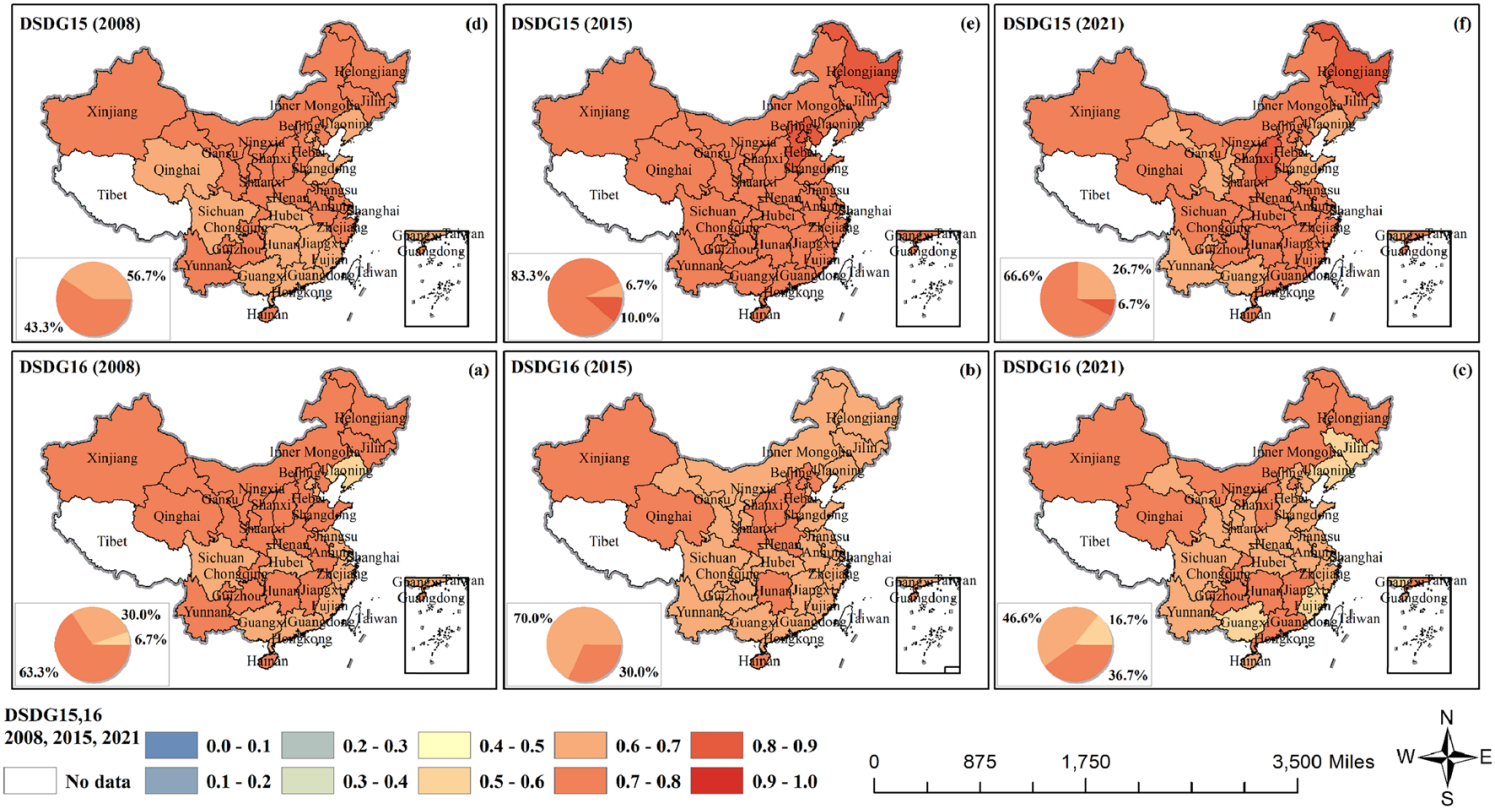
The coupling coordination degrees of GF and SDG15,16.

The CCD of GF and SDG15–16 showed a significant and synchronized decline in both 2010 and 2019 (Fig. 8 and Fig. S7). Green bonds and green loans can directly finance land restoration and conservation projects, and green finance can also encourage private and corporate investment in ecological conservation projects by promoting policies and incentives (tax incentives, loan concessions, etc.) (SDG15). The CCD between GF and SDG15 (terrestrial ecology) fluctuated up and down around 0.6 from 2008 to 2021, with the proportion of provinces achieving intermediate coordination increasing from 43.3% to 83.3% and then decreasing to 66.6%. GF also supports the establishment of peaceful and inclusive social governance structures through the promotion of transparent and accountable financial practices (SDG16). The CCD between GF and SDG16 (institutional justice) fluctuated up and down around 0.7. In 2008, 63.3% of provinces reached intermediate coordination, and this value decreased to 30.0% in 2015, with most of them concentrated in northeast China and north China. The number of provinces that reached intermediate coordination in 2021 rose to 36.7%.

### Heterogeneity analysis by regions

In this paper, the 30 provinces are divided into east, central and west regions according to the degree of economic development, and their value is the average of the coupling coordination degree of each region containing provinces. Most CCDs between GF and SDGs were in weak synergy in all 3 regions.

**Figure 9:**
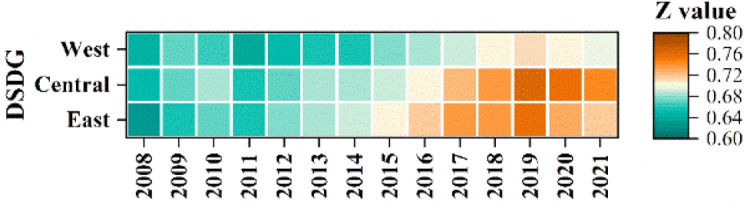
The coupling coordination degrees of GF and SDG in the East, Central and West regions. Notes: Z value means the CCD score, the same below.

The DSDG first increased and then decreased until 2011, then showed an increasing trend, and peaked in 2019 and then decreased (Fig. 9). The values in the central and eastern regions were significantly higher than those in the western region, with values higher than 0.7, while the values in the western region were mostly lower than the 0.7. The degree of coordination of the coupling of the GF with the SDG and its sub-objectives in each region was basically in weak synergy.

**Figure 10:**
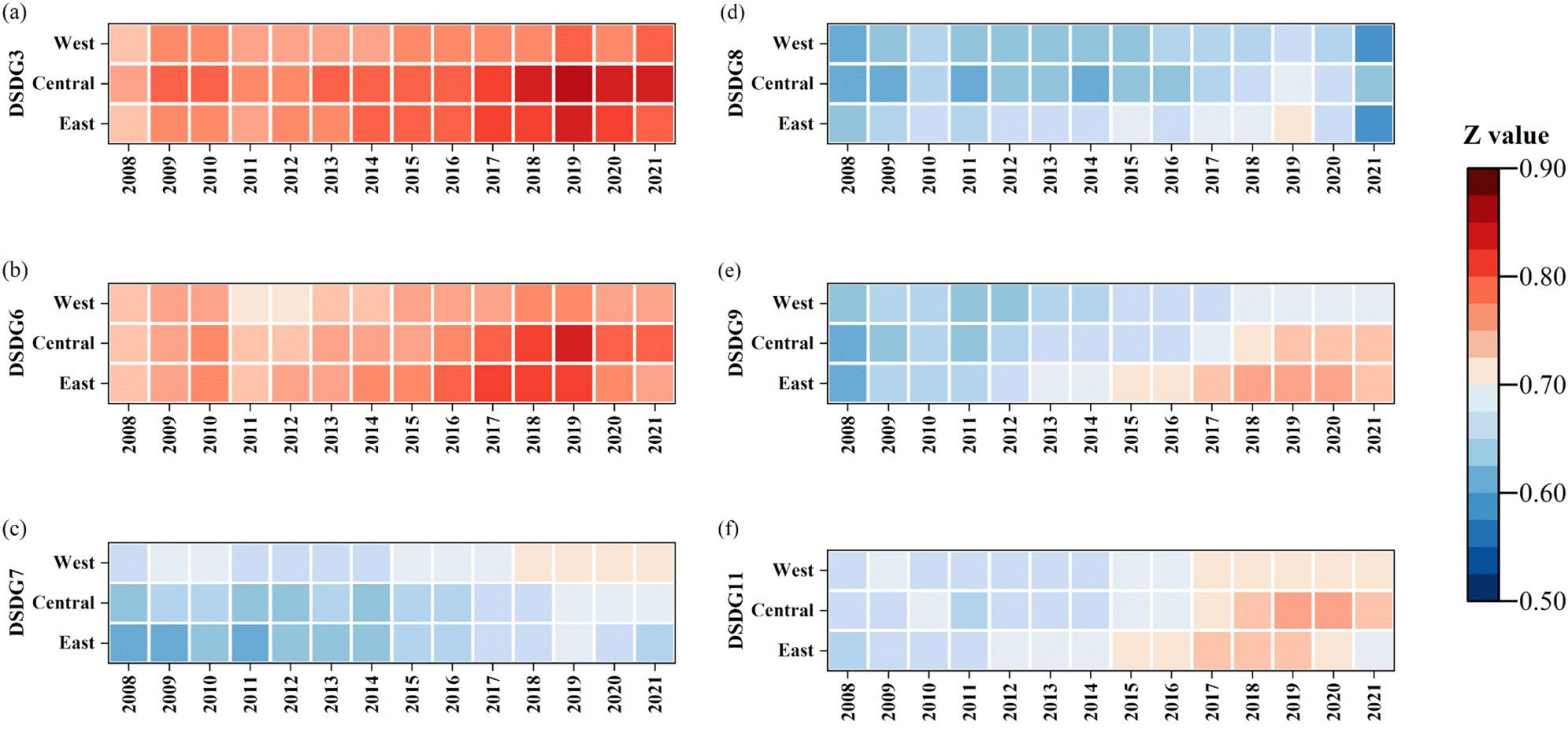
The coupling coordination degrees of GF and SDG3,6–9,11 in the East, Central and West regions.

DSDG3,6–9,11 rose and then fell until 2011, then showed an upward trend and fell after peaking in 2019 (Fig. 10 and Fig. S8). In DSDG3 (health and well-being), the eastern and central regions were significantly higher than western region, and the gap was small, with all three regions reaching intermediate coordination. The differences in DSDG6 (clean water) among the three regions were small before 2011, and the gaps have been bigger after 2011. The western region has always had the worst value. In DSDG7 (clean energy), the western region outperformed the eastern and central region, and the difference between the eastern, central and western regions gradually decreased with the growth of time, and were in primary coordination overall. DSDG8 (decent work) in the eastern region was significantly stronger than the other regions, and the difference between the central and western regions was extremely small before 2017, and gradually widened after 2017. All regions had a relatively flat trend in the period. In DSDG9 (industrial innovation), the eastern region performed the best, and the western region performed the worst, and the regional differences are more obvious. There was little difference in DSDG11 (perpetual community) among the three regions, with the eastern and central regions slightly outperforming the west, reaching intermediate coordination late in the year, and the west only reaching primary coordination.

**Figure 11:**
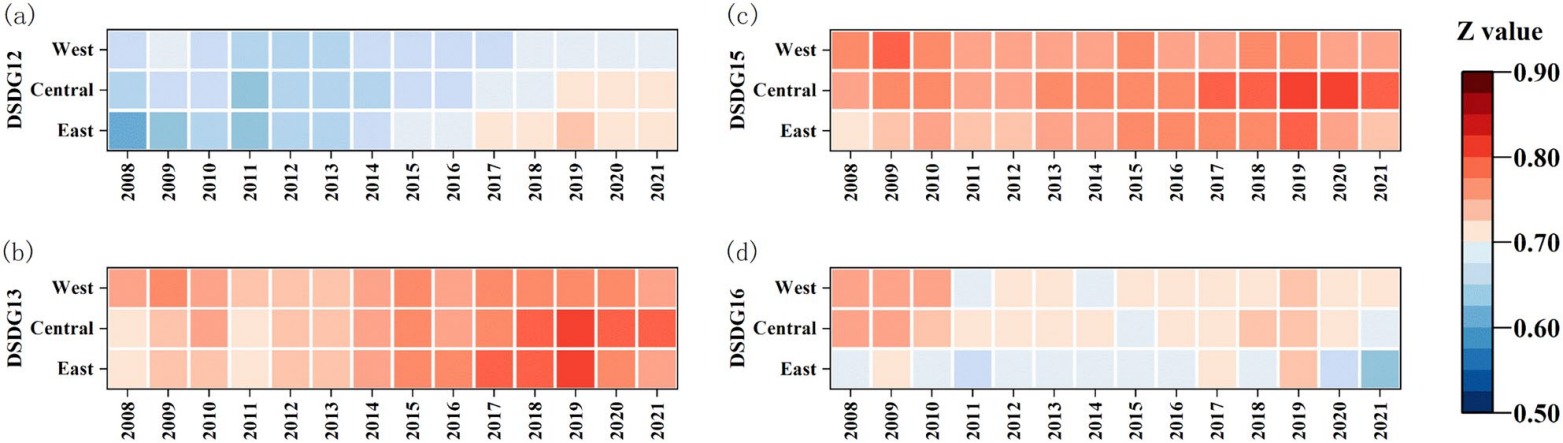
The coupling coordination degrees of GF and SDG12,13,15,16 in the East, Central and West regions.

DSDG12–12,15–16 were all increasing and then decreasing until 2011, and then showed an upward trend (Fig. 11 and Fig. S9). Before 2012, the west had the best performance in DSDG12 (perpetual supply and demand), and the east had the worst. After 2012 there was a reversal, with the west gradually declining from the best to the worst, and the east gradually moving from the worst to the best. Before 2011, the west had the best performance, and the gap between the central and the east was small in DSDG13 (climate action). After 2011, the west declined to the worst, and the gap between the central and the east was bigger. Over all, the three regions had very small differences and highly coincident fluctuation trends, and were basically in intermediate coordination. In DSDG15 (terrestrial ecology), there was less difference in the values of the three regions, with better values in the central region and worse values in the western region. All regions were in intermediate coordination. The performance of the regions in DSDG16 (institutional justice) was very similar, with a flat trend before 2019 and a significant decline after 2019, from intermediate to primary coordination.

## Discussion

This study emphasizes the coupling coordinated development degree of GF and the 11 SDGs sub-goals. Overall, the coupling coordination degree between GF and SDGs is greater than 0.40, and there is no imbalance. It indicates that China’s green finance and sustainable development are in a mutually promoting state. Green finance refers to financial activities aimed at supporting environmental improvement, addressing climate change, and resource conservation and efficient utilization in China. This highly aligns with the goals of SDG13–15 (climate change, underwater ecology, terrestrial ecology). At the same time, green finance can also indirectly or directly affect other SDGs sub-goals through industrial upgrading^36,37^, reducing energy consumption scale^23,24,38^, and carbon dioxide emissions^39,40^. There are differences in the degree of coupling and coordination between green finance and various sustainable development subgoals in different regions of China, which mainly stem from the differences in resources and endowments between the eastern, central and western regions: the eastern region includes the Yangtze River Delta (YRD), the Pearl River Delta (PRD) and the Beijing-Tianjin-Hebei (BJTH) three major economic zones, which are the most economically developed regions, with superior geographic locations, many high-end talents, and other resources, and are the "testing ground" and "demonstration zone" for reforms in many fields. It is a "testing ground" and "demonstration area" for reforms in many fields and is the most economically developed region. The central region has a large population and abundant human resources, but not much per capita resources and a fragile ecological environment. The western region, with its vast area and complex terrain, has a late history of development, little environmental pollution, and a wide gap between its level of economic development and technical management and that of the central and eastern regions, but its rich natural resources have great potential for development.

The coupling coordination degree of green finance and each sustainable development goals has three very obvious time nodes in 2011, 2012 and 2019, with evident synchronization in the development trend of the coupling coordination degree before and after these three points. After 2012, the construction of China's green financial system entered a rapid development and leapfrog stage, marked by the successive introduction of the Green Credit Guidelines (2012) and the Corporate Environmental Credit Evaluation (Trial) (2013), etc., leading to accelerated development of green finance^32^. During the same period, various sustainable development sub-goals steadily advanced in China, with the coupling coordination degree showing a clear upward trend. The outbreak of the COVID-19 pandemic in December 2019 weakened China's economic development and had a significant impact on all sectors. Green finance and sustainable development were affected differently. The national SDG6, SDG8, SDG13, and SDG15–16 declined after 2019 in the 2019–2021 period, while the averages for the remaining sub-goals increased. The epidemic brought both negative impacts and new development opportunities for the SDGs^33^. The mean value of green finance over the same period declined significantly after 2019, mainly due to the decrease in the share of energy efficiency and environmental protection expenditures. However, the epidemic only slowed down the process of achieving the SDGs^34^. As a result, the coupling of green finance with most of the SDGs decreases in CCD.

## Conclusions and policy implications

### Conclusions

In this study, we used panel data from 30 provinces (Municipalities and autonomous regions) in China from 2008 to 2021, and constructed the Coupling Coordination Degree Model to assess the degree of coupling coordinated development between the GF system and the SDGs system. We found that SDG5,7–8,10 and SDG14–16 fluctuated steadily within specific ranges, while SDG and other sub-goals show a significant upward trend. The results indicate that China has improved its SDG level over time (Xu, 2020).

GF exhibits a fluctuating upward trend, with significant declines observed in 2011 and 2020. The coupling coordination degrees between GF and SDG, as well as its sub-goals, generally show M-shaped upward trend in most regions, with many experiencing a synchronous decline in 2010–2011 and 2020. These fluctuations may be attributed to the release of green finance-related policies and the impact of the COVID-19 pandemic. In the analysis of regional heterogeneity, the eastern region performs better in SDG8–9, the central region performs better in SDG3 and SDG15, and the western region performs better in SDG7.

### Policy implications

Firstly, for policymakers, the intricate relationship between GF and the SDGs poses a challenge to traditional decision-making processes. Policymakers should adopt a comprehensive approach to formulate future policies, considering the coupling coordinated development perspective of the GF system and the SDGs system. By understanding the coupling and synergistic relationship between green finance and sustainable development, the government can develop a rational sustainable development and green finance linkage supervision and early warning mechanism. This approach enables the prediction of changes in one system from changes in the other, leading to a win–win situation. Secondly, the government should continue to develop green financial systems and address the deficiencies in the structure of the green financial system. It should also promote the development of various green financial products and markets. Additionally, enhancing the standardization of the information disclosure system and fostering open and transparent green financial markets and products can attract more private investors. The investors, in turn, will contribute to the improvement of green finance and the realization of sustainable development goals. Thirdly, according to the Global Green Finance Development Index (GGFDI) of the IFF 2021 Global Finance and Development Report, although China ranks at the forefront in terms of green finance development, there is still obvious regional heterogeneity due to factors such as the large number of ethnic groups, uneven economic development and geographic differences. When formulating policies, local governments should take into account the synergy between GF and the SDGs to facilitate the sustainable development. Different regions exhibit varying degrees of coupling and coordination between green finance and sustainable development goals and their sub-goals. Regions with low coupling and coordination should actively learn from the experience of regions with high coupling and coordination. Subsequently, local governments should adjust their relevant development policies according to the actual situation of the region. They should develop a variety of coordinated development policies on green finance and sustainable development tailored to local conditions.

Some limitations are reflected: (1) The indicator system established in this paper maybe comprehensive enough to accurately reflect the level of sustainable development and green finance in China. Obtaining more accurate and comprehensive data could lead to more instructive conclusions about the real situation. (2) The coupling and synergy between green finance and SDGs are not explored in depth, and the channels through which green finance promotes SDGs are not thoroughly explored. Future research can be expanded from the following aspects: (1) Investigate the specific channels through which green finance promotes the coupling coordinated development of the SDGs sub-goals. (2) Explore the concepts of intercoupling, pericoupling, and telecoupling within the framework of metacoupling^35,41^ regarding the relationship between green finance and SDGs sub-goals. (3) Systematically analyze the interaction between green finance and different Sustainable Development Goals, and formulate policy for coupling coordinated development of green finance and sustainable development from the metacoupling perspective.

### Supplementary Information

Supplementary Information.

## Data Availability

The data supporting the findings of this study are available from Natural Science Research Project of Guizhou Provincial Department of Education. However, restrictions apply to the availability of these data, as they were used under license for the current study and are therefore not publicly available. Nevertheless, the data can be obtained from the authors upon reasonable request and with the permission of Natural Science Research Project of Guizhou Provincial Department of Education. For any additional data relevant to the manuscript, interested parties should reach out to the corresponding author.
