# Supplementary material for: Assessment of the coupling coordination relationship between the green financial system and the sustainable development system across China
Source: Sci Rep. 2024 May 21;14:11534. doi: 10.1038/s41598-024-62471-8 (PMC11109143; doi:10.1038/s41598-024-62471-8)
Supplement: Supplementary file 1 — Supplementary Information. [file 41598_2024_62471_MOESM1_ESM.docx]

## Supplementary Tables

Supplementary Table 1 Indicators of sustainable development

| Sustainable development goals (SDGs) | Measurement indicators | Attribute |
| --- | --- | --- |
| SDG1  End poverty in all its forms everywhere | Proportion of rainfall in the reservoir capacity | + |
|  | Economic loss/GDP | - |
|  | Affected population | - |
|  | Proportion of the education, healthcare and social security spending in the fiscal expenditure budget | + |
|  | Coverage of basic endowment insurance | + |
|  | Coverage of unemployment insurance | + |
|  | Coverage of basic medical insurance | + |
| SDG2 End hunger, achieve food security and improved nutrition and promote sustainable agriculture | Gross output of animal husbandry | + |
|  | Gross output of forestry | + |
|  | Gross output of agriculture | + |
|  | Soil erosion control area | + |
|  | Moderate and severe malnutrition  among children under five years of age | - |
|  | Grain yield per unit area | + |
| SDG3  Ensure healthy lives, and promote well-being for all at all ages | AIDS incidence per 100,000 people | - |
|  | Hepatitis B incidence per 100,000 people | - |
|  | Malaria incidence per 100,000 people | - |
|  | TB incidence per 100,000 people | - |
|  | Date rate due to road traffic injuries (per 10,000 people) | - |
|  | Perinatal mortality rate | - |
|  | Maternal mortality rate | - |
|  | The number of deaths in traffic accidents | - |
|  | Number of health workers per 10,000 people | + |
|  | Number of medical and health institutions | + |
|  | Number of beds in medical and health institutions | + |
| SDG4 Ensure inclusive and equitable quality education and promote lifelong learning opportunities for all | Teacher-student ratio in primary schools (number of teachers=1) | - |
|  | Teacher-student ratio in secondary schools (number of teachers=1) | - |
|  | Teacher-student ratio in regular institutions of higher learning (number of teachers=1) | - |
|  | The proportion of the population aged six years and older who have never attended school | - |
|  | Male–female ratio who have never attended school (female=100) | + |
|  | Proportion of government expenditure on education in total government expenditure | + |
|  | Number of students enrolled in special education schools (per 10,000 people) | + |
|  | Proportion of the population aged 15 years and older who are illiterate | - |
| SDG5 Achieve gender equality and empower all women and girls | Proportion of female representatives in the workers’ congress | + |
|  | Proportion of employed women | + |
|  | Male–female ratio in the population (female=100) | - |
|  | Male–female ratio in the illiterate population (female=100) | + |
|  | Male–female ratio in the population that has received higher education (female=100) | - |
| SGD6 Ensure availability and sustainable management of water and sanitation for all | Water-use efficiency (water consumption per GDP) | - |
|  | Urban water popularizing rate | + |
|  | Total water supply | + |
|  | Daily urban sewage treatment capacity | + |
|  | Chemical oxygen demand emissions | - |
| SDG7 Ensure access to affordable, reliable, sustainable and modern energy for all | Energy intensity | + |
|  | Waste water discharge | - |
|  | Per capita electricity consumption | + |
|  | Proportion of thermal power generation in the total power generation | - |
|  | Per capita water consumption | + |
| SDG8 Promote sustained, inclusive, and sustainable economic growth, full and productive employment and decent work for all | Proportion of international tourism revenue in the GDP | + |
|  | Annual growth rate of per capita GDP | + |
|  | Registered urban unemployment rate | - |
|  | Average salary of employed population in urban workplaces | + |
| SDG9 Build resilient infrastructure, promote inclusive and sustainable industrialization and foster innovation | Popularizing rate of mobile phones (phone/100 people) | + |
|  | Patent applications granted per 10,000 people | + |
|  | Number of R&D personnel per 10,000 people | + |
|  | Volume of freight traffic | + |
|  | Proportion of manufacturing employment in the employment of urban workplaces | - |
|  | CO2 emissions/GDP | - |
|  | RD funding investment intensity | + |
|  | Industrial added value (as a percentage of GDP) | - |
| SDG10 Reduce inequality within and among countries | Proportion of wages in GDP | + |
|  | Ratio of per capita disposable income  between rural and urban areas | + |
|  | Growth rate of disposable income of rural residents | + |
|  | Proportion of social security and  employment expenditures in fiscal expenditure budget | + |
|  | Male–female ratio in the population (female =100) | - |
| SDG11 Make cities and human settlements inclusive, safe, resilient and sustainable | Number of public transport vehicles  per 10,000 people | + |
|  | Passenger transport capacity | + |
|  | Per capita park and green space area | + |
|  | Number of public toilets per 10,000 people | + |
|  | Number of hospitals | + |
|  | Volume of urban household garbage clearance | - |
|  | Smoke and dust emissions | - |
|  | SO2 emissions | - |
|  | Road cleaning area | + |
|  | Daily urban sewage treatment capacity | + |
| SDG12  Ensure sustainable consumption and production patterns | Per capita consumption expenditure of all residents | + |
|  | Per capita water consumption | + |
|  | Per capita natural gas consumption | + |
|  | Per capita waste water generated | - |
|  | Per capital industrial solid waste generated | - |
|  | Number of invention patents granted | + |
| SDG13 Take urgent action to combat climate change and its impacts | People affected by natural disasters | - |
|  | Carbon emissions | - |
|  | Environment protection expenditure | + |
| SDG14 Conserve and sustainably use the oceans, seas, and marine resources for sustainable development | Researchers in the field of marine research | + |
|  | Proportion of gross ocean product | - |
|  | R&D programs of marine research and  development agencies | + |
|  | Direct economic loss of storm surge disaster | - |
|  | Marine fishing output | - |
|  | Mariculture output | - |
| SDG15 Protect, restore, and promote sustainable use of terrestrial ecosystems, sustainably manage forests, combat desertification, and halt and reverse land degradation, and halt biodiversity loss | Forest coverage | + |
|  | Area of artificial afforestation | + |
|  | Area of fire-affected forests | - |
|  | Proportion of direct economic loss of  natural disasters in GDP | - |
|  | Proportion of environment protection expenditure in total fiscal expenditure | + |
| SDG16 Promote peaceful and inclusive societies for sustainable development, provide access to justice for all and build effective, accountable and inclusive institutions at all levels | Birth rate | + |
|  | Proportion of public service expenditure in the fiscal expenditure budget | + |
|  | Crime rate | - |
|  | Proportion of public servants in the total population | + |
| SDG17 Strengthen the means of implementation and revitalize the Global Partnership for Sustainable Development | Internet broadband access port | + |
|  | Proportion of tax revenue in the GDP | - |
|  | Proportion of health and education expenditure in GDP | + |
|  | Proportion of social security and employment expenditure in fiscal expenditure budget | + |
|  | Proportion of total foreign investment in GDP | + |
|  | Proportion of fiscal revenue in GDP | - |

Supplementary Table 2 Indicators of green finance

| Primary indicator | Secondary indicator | Tertiary indicator | Definition of indicator | Attribute |
| --- | --- | --- | --- | --- |
| Green finance  (GF) | Green credit  (credit) | Proportion of interest expenditure of energy-intensive enterprises | Interest expenditure of energy-intensive enterprises above the designated size/interest expenditure of industrial enterprises above designated size | - |
|  | Green security  (security) | Proportion of market value of energy-intensive industries | Market value of A shares of energy-intensive industries/total market value of A shares | - |
|  | Green investment  (investment) | Proportion of energy conservation and environment protection expenditure | Fiscal energy conservation and environment protection expenditure/total fiscal expenditure | + |
|  | Green insurance  (insurance) | Proportion of agricultural insurance | Agricultural insurance revenue/property insurance revenue | + |
|  |  | Claim ratio of agricultural insurance | Agricultural insurance expenditure/agricultural insurance revenue | + |

Notes: Energy-intensive industries in the green credit include petroleum processing industry, coking and nuclear fuel processing industry, chemical raw materials and chemical products manufacturing industry, non-metallic mineral products industry, ferrous metal smelting and rolling processing industry, non-ferrous metal metallurgy and rolling processing industry, electricity and heat production and supply industry.

|  |
| --- |
| Fig. S1 Performance on GF in China |

|  |
| --- |
| Fig. S2 Performance on SDG in China |

|  |
| --- |
| Fig. S3 Performance on SDG1-9 in China |

|  |
| --- |
| Fig. S4 Performance on SDG10-17,14-11 in China |
| Notes: (1) SDG14 is calculated by 30 provinces, SDG14-11 is calculated by 11 provinces; (2) The rightmost legend is for SDG10-17, the legend next to SDG14-11 is only for SDG14-11. |
|  |
| Fig. S5 Performance on the CCD of GF and SDG in China |
|  |
| Fig. S6 Performance on the CCD of GF and SDG3,6-9,11 in China |
|  |
| Fig. S7 Performance on the CCD of GF and SDG12-16 in China |
| Notes: (1) SDG14 is calculated by 11 provinces; (2) The rightmost legend is for SDG12,13,15,16, the legend next to SDG14 is only for SDG14. |
|  |
|  |
| Fig. S8 Performance on the CCD of GF and SDG3,6-9,11 in Eastern, Central and Western China |
|  |
| Fig. S9 Performance on the CCD of GF and SDG12,13,15,16 in Eastern, Central and Western China |
